# LINC00662 promotes melanoma progression by competitively binding miR-107 and activating the β-catenin signaling pathway

**DOI:** 10.7150/ijms.84072

**Published:** 2024-01-01

**Authors:** Mengmeng Luo, Rui Lei, Qingfang Zhao, Yichen Shen, Zhigang He, Jinghong Xu

**Affiliations:** Department of Plastic Surgery, The First Affiliated Hospital, School of Medicine, Zhejiang University; Hangzhou, China.

**Keywords:** Melanoma, LINC00662, MiR-107, POU3F2

## Abstract

Melanoma is a highly malignant tumor in the body. Long non-coding RNAs (lncRNAs) have been reported to be involved in the development of various tumors. Emerging evidence demonstrates the critical role of lncRNAs in melanoma development. In this study, we aimed to investigate the expression, biological function and regulatory mechanism of LINC00662 in melanomas. First, we found that LINC00662 was up-regulated in melanoma tissues and cell lines. High expression of LINC00662 in melanomas was associated with a poor patient prognosis. Silencing of LINC00662 suppressed the proliferation, migration, and invasion of melanoma cells *in vitro* and *in vivo*, while overexpression of LINC00662 promoted melanoma cell proliferation *in vitro*. Bioinformatics analysis, dual-luciferase assay, and RIP assay confirmed that LINC00662 competitively regulated miR-107. Silencing of LINC00662 upregulated miR-107 expression in a melanoma cell line. Inhibition of miR-107 significantly reversed the inhibitory effect of LINC00662 silencing on cell proliferation and migration. Furthermore, POU3F2 was validated as a downstream target of LINC00662/miR107 and was downregulated when LINC00662 was silenced. Overexpressing POU3F2 attenuated the effect of si-LINC00662 on cellular functions. In addition, the results also showed that the β-catenin pathway was involved in a si-LINC00662-induced function in melanoma. Overall, our results confirmed that LINC00662 promoted melanoma progression by sponging miR107 and inducing POU3F2, highlighting the mechanism of the LINC00662/miR-107/POU3F2 axis in melanoma cell proliferation and invasion.

## Introduction

Melanoma is the most harmful skin disease in western countries and results in 10,000 deaths each year 1. In China, its malignancy is ranked at the forefront of a variety of tumors, and the number of cases each year is also rising sharply 2, 3. Melanoma is characterized by a high degree of malignancy and remote metastasis. Once metastasis occurs, the survival prognosis is quite poor, with a 3-year survival rate of less than 10% and a median survival time of about 8 months 4. If melanoma can be found early, the outcome is promising, it generally can be treated by surgery, the use of chemotherapy, and other means, and the cure rate is up to 90% 5. However, clinical melanoma patients are often found in the middle and late stages of the disease, patients at this time often develop resistance to chemotherapy or radiotherapy, and the tumor is prone to metastasis 6. Therefore, early detection of melanoma is a major way of improving patient survival, which requires us to further understand the occurrence and development of the disease.

In recent years, the study of long noncoding RNA (lncRNA) has become a hot topic in many fields, especially in oncology 7-9. Dissimilar to general microRNA or mRNA, lncRNA is an RNA of over 200 nt and is not capable of encoding proteins 10, 11. Previous studies have shown that lncRNAs frequently act as tumor promotors or tumor suppressors in many malignant tumors, including melanoma, and play important roles in cell proliferation, cell cycle, migration, and invasion. For instance, Juan et al. found that, in gastric cancer, lncRNA MYLK-AS1 could act as a tumor promoter 12. Sometimes, a single lncRNA can play an oncogene role in multiple tumors, BCYRN1 acts as an oncogene in esophageal squamous cancer, gastric cancer, colorectal cancer, hepatocellular carcinoma, and non-small cell lung cancer 13. In addition, miR99AHG has been shown to act as a tumor suppressor in lung adenocarcinoma 14. P53-targeted lncRNA ST7-AS1 has also been shown to play a tumor suppressor role in glioma by influencing the Wnt/β-catenin signaling pathway 15.

The functional mechanisms of lncRNA are complex and diverse. One of the important mechanisms of cytoplasmic lncRNA is to competitively bind to common microRNAs (miRNAs) and regulate the expression of their target genes. These lncRNAs are classified as competing endogenous RNAs (ceRNAs). However, research into lncRNA in melanoma is still in its infancy, and the specific functions and mechanisms of most lncRNAs are still not fully clarified.

LINC00662 is 2,085 nucleotides in length, contains three exons, and is mainly located in the cytoplasm. LINC00662 has been reported in many different tumor types and is generally considered to be an oncogene. For example, in colon cancer, LINC00662 promotes tumor growth and metastasis by sponging miR-340-5p 16 and plays an oncogenic role in bladder cancer by sponging miR-199a-5p 17. LINC00662 has also been reported to be oncogenic in melanoma by sponging miR-890 18. Notably, a previous study has shown that miR-107 was a novel tumor suppressor targeting POU3F2 in melanoma 19, and our bioinformatics analysis predicted that miR-107 could be a target of LINC00662. Notwithstanding the aforementioned research, whether LINC00662 affects the biological behavior of melanoma cells through regulating miR-107 remains to be determined, and the specific regulatory mechanism still needs further study.

Therefore, in the present study, we focused on the interaction between LINC00662 and miR-107 in the progression of melanoma. We hypothesized that LINC0062 might be involved in the development of melanoma via regulating the miR107/POU3F2 axis. We aimed to demonstrate that LINC00662 may regulate POU3F2 expression in melanoma by competitively binding miR-107 in this study.

## Materials and Methods

### Cell culture

The melanoma cell lines SK-MEL-28 and A375 were purchased from the Institute of Biochemistry and Cell Biology of the Chinese Academy of Sciences (Shanghai, China). The melanoma cell lines M21 and SK-MEL-110 were kindly provided by the Central Laboratory, Tongji University Affiliated People's 10^th^ Hospital. The normal human epidermal melanin cell line, HEMa-LP, was purchased from Thermo Fisher Scientific (Shanghai, China). All cells were cultured in high glucose Dulbecco's Modified Eagle's medium (DMEM; Hyclone, Logan, UT, USA) containing 10% fetal bovine serum (FBS; Gibco-BRL Life Technologies, Paisley, UK) and 1% penicillin/streptomycin (Invitrogen, Carlsbad, CA, USA) at 37°C in a humidified incubator with 5% CO_2_.

### Cell transfection

All cell transfection experiments were performed in 6-well plates at 37°C in 5% CO_2_. The day before transfection, SK-MEL-28 and A375 cells were seeded into 6-well plates at a certain density, 1 × 10^5^ for microRNA (or its inhibitors) and siRNA transfection and 2 × 10^5^ for plasmid transfection. After 24 hours of incubation, different small RNAs (si-NC, si-LINC00662, miR-NC, miR-107 mimics, and miR-107 inhibitor) or plasmids (pcDNA3.1-vector, pcDNA3.1-LINC00662, pcDNA3.1-POU3F2, pmirGLO, pmirGLO-LINC00662-WT, and pmirGLO-LINC00662-mut) were transfected into SK-MEL-28 and A375 cells by using Lipofectamine 2000 (Invitrogen) according to the manufacturer's instructions. The final transfection concentration for small RNA was 20 nM and the amount of plasmid used was 2.5 µg/well. After an additional 48 hours of incubation, the cells were collected for use in subsequent studies. The microRNAs, siRNAs, and plasmids were purchased from RiboBio Co., Ltd. (Guangzhou, China), and the sequences are shown in Table S1.

### RNA isolation and real-time quantitative PCR (RT-qPCR) analysis

Total RNA in different treatment groups of cells was isolated by using TRIzol reagent (Invitrogen) according to the manufacturer's protocol, and we tested the RNA purity using an ultraviolet spectrophotometer. The integrity of RNA was determined using agarose gel electrophoresis, and the qualified RNA was used for subsequent experiments. First-stand cDNA was generated using a PrimeScript RT Reagent Kit (TaKaRa, Dalian, China). A total of 1 μg RNA was used for each sample, and the reaction system was 20 µL, after cDNA synthesis. We diluted it 1:19 with ddH_2_O, and then by using different primers. A SYBR Premix Ex Taq (TaKaRa) kit was used to detect the expressions of LINC00662, miR-107, and POU3F2. Ct values were determined in triplicate, and the results were analyzed using an ABI 7500 Fast Real-Time PCR System (Applied Biosystems, Foster City, CA, USA). The relative gene expression was calculated by the 2^-ΔΔCt^ method using different controls (U6 for miR-107 and GAPDH for LINC00662 and POU3F2, respectively). The primer sequences for PCR are listed in Table S1.

### Cell cycle analysis

A total of 1 × 10^5^ cells were seeded into 6-well plates, and sh-LINC00662 and its control were transfected for 48 hours. The harvested cells were transferred to a 1.5 mL EP tube for centrifugation (1,000 rpm, 5 min). After the supernatant was discarded, 1 mL of pre-cooled PBS was used for two sequential washings. One mL pre-cooled 70% ethanol was then added, gently and mixed, and fixed at 4℃ overnight. The next day, cold PBS was used to wash the cells, followed by staining with PI/RNase (BD Pharmingen, San Jose, CA, USA) for 30 min at room temperature in the dark. Finally, 1 × 10^5^ cells were detected by flow cytometry at different cell cycle phases (G0, G1, S, and G2).

### Cell proliferation analysis

Cell proliferation was measured with a Cell Counting Kit-8 (CCK-8; Dojindo, Tokyo, Japan). We seeded 2,000 cells into each 96-well plate. At four time points on day 1, day 2, day 3, day 4, and day 5, the CCK-8 assay solution (10 μL) was added to each well and incubated for another 1.5 hours. The absorbance values for each well at 450 nm were measured with an enzyme immunoassay analyzer (Thermo Fisher Scientific).

For the colony formation assays, the harvested cell suspension was diluted by gradient multiples, and adjusted to a concentration to 250 cells/mL. A 2 mL cell suspension was then seeded into 6-well plates and incubated for 7-14 days. When the number of monoclonal cell colonies reached 100, the colonies were washed with PBS, fixed with 95% ethanol for 10 min, and stained with 0.1% Crystal Violet (Sigma-Aldrich, St. Louis, MO, USA) for 15 min. The cells were then washed with water and counted.

### Wound healing assay

Cells (1 × 10^5^) were seeded into a 6-well plate and when the cell fusion reached 95%, the cells were scratched with a 200 µL pipette tip. Then, the FBS percentage in the medium was reduced to 2%. The wound was then imaged at 0 hours and 36 hours. The migration capacity of cells was evaluated by calculating the area of cell healing, using the formula: [area (0 hours) - area (36 hours)]/area (0 hours), using ImageJ software (National Institutes of Health, Bethesda, MD, USA).

### Transwell invasion assay

Cell invasion assays were performed using Transwell inserts (8-μm pore size; Corning, Cambridge, MA, USA) coated with Matrigel (BD Biosciences, San Jose, CA, USA). Cells (2 × 10^4^) cultured in 200 μL serum-free DMEM (Hyclone,) were added to the upper chamber. Complete medium (600 μL) containing 20% FBS was added to the lower chamber. Following 36 hours incubation at 37°C, cells adhering to the lower surface of the Transwell membrane were fixed with 4% paraformaldehyde (PFA), stained with 0.1% Crystal Violet, and rinsed with PBS. Cell counts were then conducted using phase contrast microscopy (Leica, Wetzlar, Germany) in five random fields, and the numbers of invaded cells were analyzed.

### Xenograft mouse model

The DNA encoding short hairpin RNA (shRNA) specifically targeting LINC00662 (sh-LINC00662) was designed by Takara (Dalian, China). To obtain LINC00662 stably silenced melanoma cells, shRNA-NC, and shRNA-LINC were transfected into A375 cells using a lentiviral vector packaging system. A375 cells (5 × 10^6^) expressing shRNA (shLINC00662 or shCtrol), as indicated, were subcutaneously (SC) injected into 6-week-old athymic nude mice (Bikai, Shanghai, China). Tumor size was measured every 4 days, and the tumor volume was calculated as 0.5 × length × width^2^. Mice were euthanized using excess CO_2_ at 30 days after injection, and the tumor was weighed after sampling.

### The luciferase reporter assay

Microcode bioinformatics tools, RNA22 and mircode database, were used to find the potential binding sequences of miR-107 and LINC00662. The wild type (wt) and mutant (mut) fragments of LINC00662 were cloned into a pGLO vector (GenScript, Nanjing, China) to construct WT-LINC00662 and Mut-LINC00662 plasmids. Cells were co-transfected with miR-107 mimic or its NC and corresponding reporter plasmids using Lipofectamine 2000 (Invitrogen). The luciferase activity was measured 48 hours later using a Dual-Luciferase Reporter Gene Assay Kit (Promega, Madison, WI, USA) following the provided protocol. Mutations in the putative binding sites of LINC00662 were made using a Quick-Change Site-Directed Mutagenesis kit (Agilent Technologies, Santa Clara, CA, USA).

### RNA immunoprecipitation (RIP) assay

RIP was performed using a Magna RIP RNA-Binding Protein Immunoprecipitation kit (Millipore, Bedford, MA, USA) according to the manufacturer's instructions. Briefly, cells were transfected with miR-107 mimics, microRNA NC mimics, and LINC00662, then they were resuspended and lysed in lysis buffer (50 mM Tris-HCl, pH 7.4), 150 mM NaCl, 1% NP40, and 0.5% sodium deoxycholate). Simultaneously, protein A/G magnetic beads were vortexed and resuspended in 500 μL RIP wash buffer (20 mM Tris-HCl, pH 8.1), 150 mM NaCl, 1% Triton X-100, and 0.1% SDS). Next, cell lysates were centrifuged at 4a for 10 min and the supernatant was added to the resuspended magnetic beads for antibody incubation with anti-Ago2 (ab186733; Abcam, Cambridge, UK) or anti-IgG (ab133470, Abcam). Then the suspension was placed in a vertical mixer at 4℃ overnight. Samples were centrifuged briefly to collect precipitate at the bottom of the tube, which was further digested with Proteinase K (Applied Biosystems) to extract the RNA. Lastly, RT-qPCR assays were used to determine the relative enrichment of LINC00662 mRNA.

### Western blot analysis

We collected the indicated cells using a disposable cell scraper and lysed them in lysis buffer containing PMSF and cocktail inhibitors (radioimmunoprecipitation assay). The total protein was measured with a BCA Protein Quantitation kit. Equivalent amounts of total protein extract were separated by 6%-12% SDS-PAGE gels and transferred to polyvinylidene difluoride membranes (Bio-Rad, Hercules, CA, USA). Blots were then blocked with 5% skimmed milk for 120 min and membranes were incubated overnight at 4°C with primary antibodies against POU3F2 (ab243045, Abcam), E-cadherin [14472S; Cell Signaling Technologies (CST), Danvers, MA, USA], vimentin (5741T, CST), GSK3β (12456S, CST), β-catenin (8480T, CST), and GAPDH (ab8245, Abcam). On the second day, the membranes were incubated with horseradish peroxidase (HRP)-conjugated secondary antibody (7074S,7076S, CST) at 37°C for 60 min. Protein bands were visualized with enhanced chemiluminescence (ECL kit, Millipore, USA) and analyzed with Bio-Rad Imaging Systems (Bio-Rad).

### Statistical analysis

Statistical analyses were performed using SPSS version 21.0 (SPSS Inc., Chicago, IL, USA). All data are presented as mean ± standard deviation (SD) of at least three independent experiments. Statistical significance is shown as ^**^p < 0.01 or ^*^p < 0.05.

## Results

### LINC00662 was highly expressed in melanoma tissues and cells

**Figure 1:**
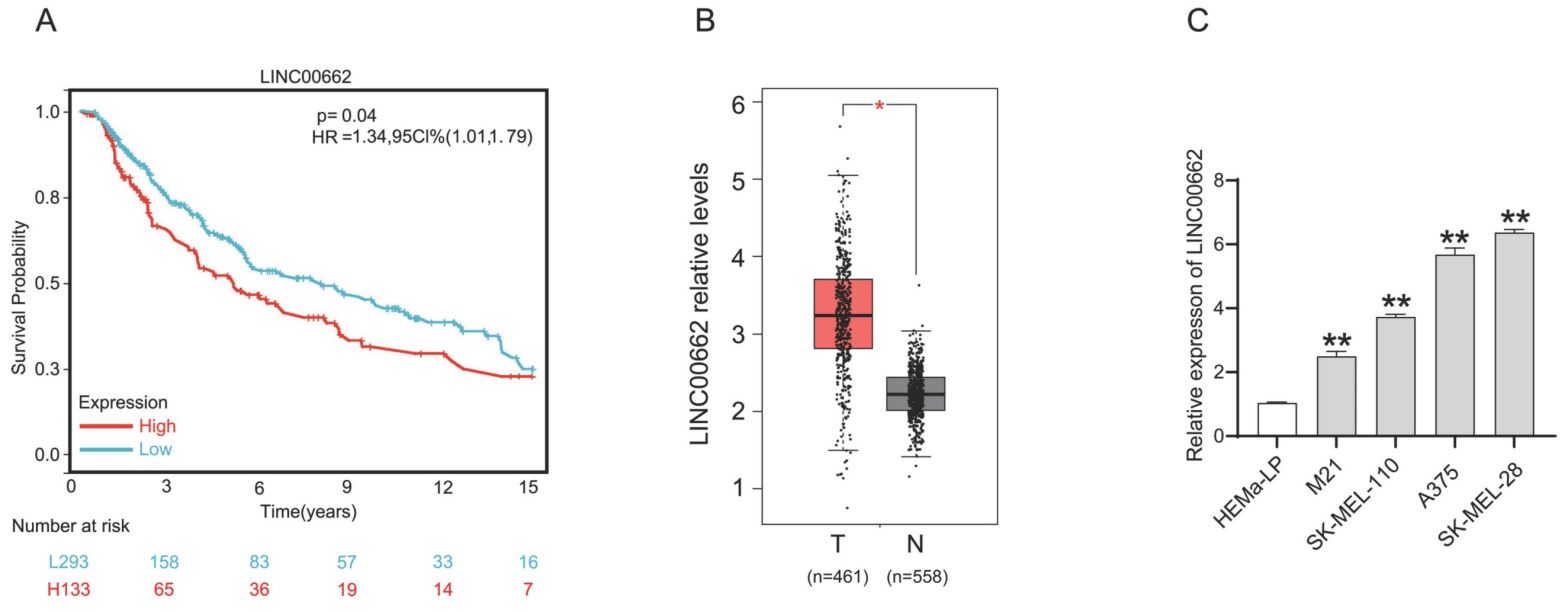
** LINC00662 was highly expressed in melanoma tissues and cells.** (A) The overall survival of LINC00662 in melanoma. (B)The expression level of LINC00662 in melanoma tissues and normal epidermal tissues. (C) Relative expression of LINC00662 in melanoma cell lines (M21, sk-mel-110, A375 and sk-mel-28) and a normal human epidermal melanin cell line (HEMa-LP). *P<0.05, **P<0.01.

We determined the expression level of LINC00662 in melanoma patients in The Cancer Genome Atlas (TCGA) database. As shown in Figure 1 A, Kaplan-Meier survival analysis demonstrated that melanoma patients with high LINC00662 expression had significantly reduced overall survival (OS). LINC00662 expression analysis was performed using the gene count output from the GEPIA database 20, and as expected, we found that the expression of LINC00662 was significantly higher in melanoma tissue than in normal epidermal tissue (Figure 1B). Moreover, real-time qPCR was used to investigate the expression of LINC00662 in melanoma cell lines (M21, SK-MEL-110, SK-MEL-28, and A375) and a normal human epidermal melanin cell line (HEMa-LP). The expression of LINC00662 was markedly higher in the melanoma cells, compared with the HEMa-LP cells (Figure 1C). The SK-MEL-28 and A375 cell lines were selected for subsequent experiments due to the relatively high expression of LINC00662.

### LINC00662 silencing inhibited melanoma cell proliferation, migration, and invasion *in vitro*

**Figure 2:**
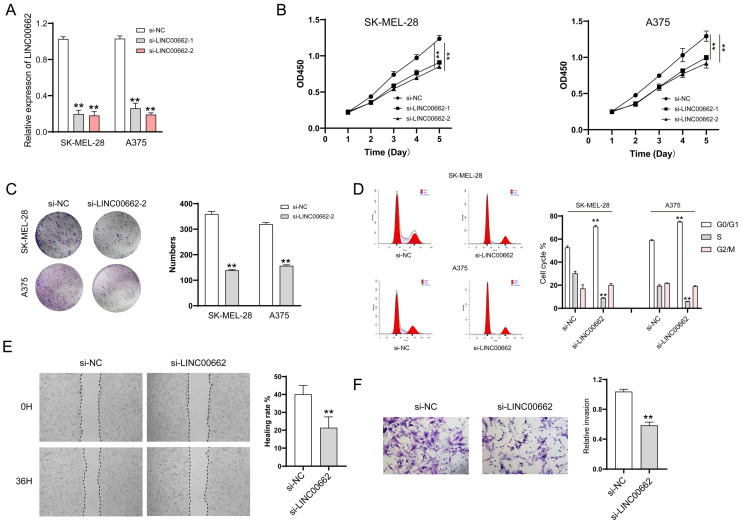
LINC00662 silencing inhibited melanoma cell proliferation, migration and invasion in vitro. (A) si-LINC00662 transfection significantly decreased the expression of LINC00662 compared with the control group in SK-MEL-28 and A375 cells. (B-C) CCK-8 and colony formation assays were conducted to detect cell proliferation when LINC00662 was silenced. (D) Flow cytometer was used to analyze the transfected cells at different cell cycle phase (G0/G1, S, and G2/M). (E-F) Wound healing (magnification, x100) and Transwell assays (magnification, x200) were used to measure the cell motility and invasiveness of SK-MEL-28 cells. **P<0.01.

For the cell function study, we first decreased the expression of LINC00662 by transfecting si-RNA. The knockdown efficiency was verified by RT-qPCR, and the results showed that si-LINC00662 transfection significantly decreased LINC00662 expression compared with the control group in SK-MEL-28 and A375 cells (Figure 2A). After silencing the expression of LINC00662 in SK-MEL-28 and A375 cells, CCK-8 and colony formation assays were performed. Cell proliferation was significantly reduced in the si-LINC00662 cells compared to the si-NC cells (Figure 2B and C). Flow cytometry assay was also used to detect the cell cycles, as shown in Figure 2D. The cell distribution was significantly changed due to silencing LINC00662. The G0/G1 phase in SK-MEL-28 cells was increased after silencing LINC00662 and the S phase was decreased, similar changes have been observed in A375. Additionally, we explored cell motility and invasiveness of SK-MEL-28 cells by using wound healing and Transwell assays, and the results suggested that after silencing LINC00662, cell migration and invasion were inhibited (Figure 2E and F). Next, we also overexpressed LINC00662 in SK-MEL-28 and A375 cells. CCK-8 assays demonstrated that overexpression of LINC00662 slightly promoted cell proliferation, but this effect was not significant (Figure S1). The possible reason was that background expressions of LINC00662 in SK-MEL-28 and A375 cells were too high to detect changes in cell proliferation. Thus, we overexpressed LINC00662 in M21 cells (relatively low LINC00662-expressing cells). The results showed that cell proliferation was significantly increased due to LINC00662 overexpression (Figure S1). Taken together, the above results confirmed that LINC00662 acted as an oncogene in melanomas.

### LINC00662 silencing inhibited tumor tumorigenicity *in vivo*

**Figure 3:**
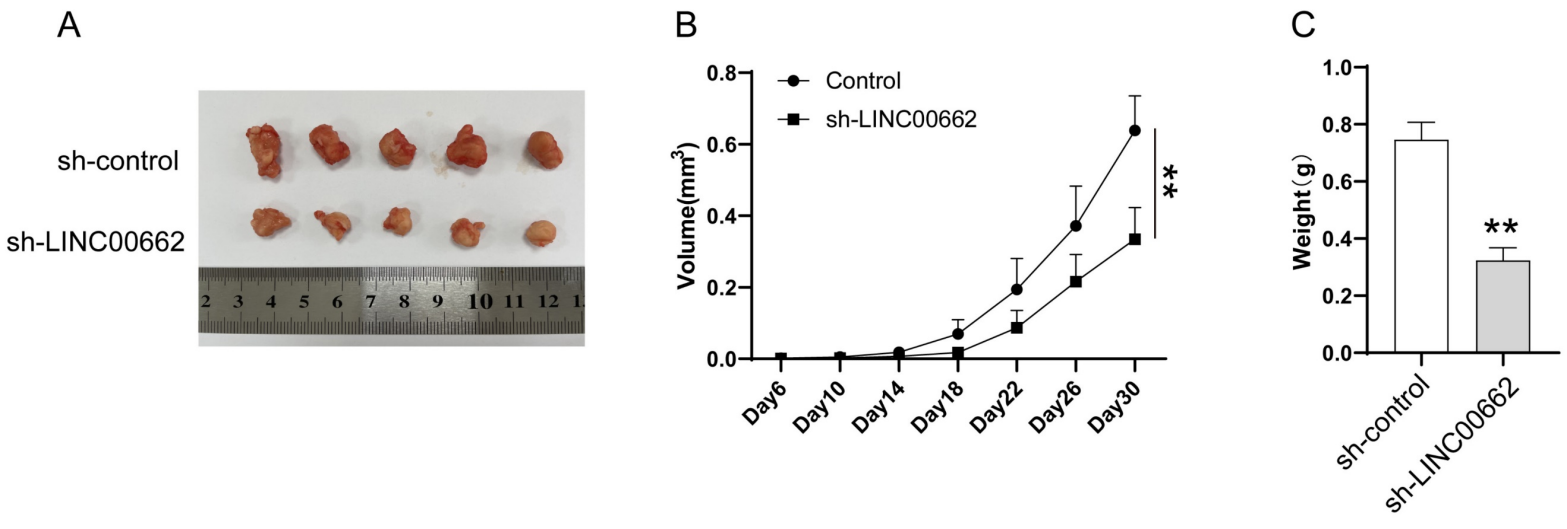
LINC00662 silencing inhibited tumor tumorigenicity in vivo. (A) Representative photos of xenografts. (B) Tumor growth curve in melanoma cell xenografts. (C) The tumor weight was measured at 30 days after injection **p<0.01.

We further studied LINC00662 using animal models, *in vivo* assays were conducted in a xenograft tumor model. Sh-LINC00662 or sh-NC was transfected into A375 cells and subsequently xenografted into nude mice, and the result showed that sh-LINC00662 could suppress tumor growth (Figure 3A). The tumor weight and tumor volume of the sh-LINC00662 group were much lighter and smaller than in the sh-NC group (Figure 3B and C).

### miR-107 was an essential downstream target of LINC00662 in melanomas

**Figure 4:**
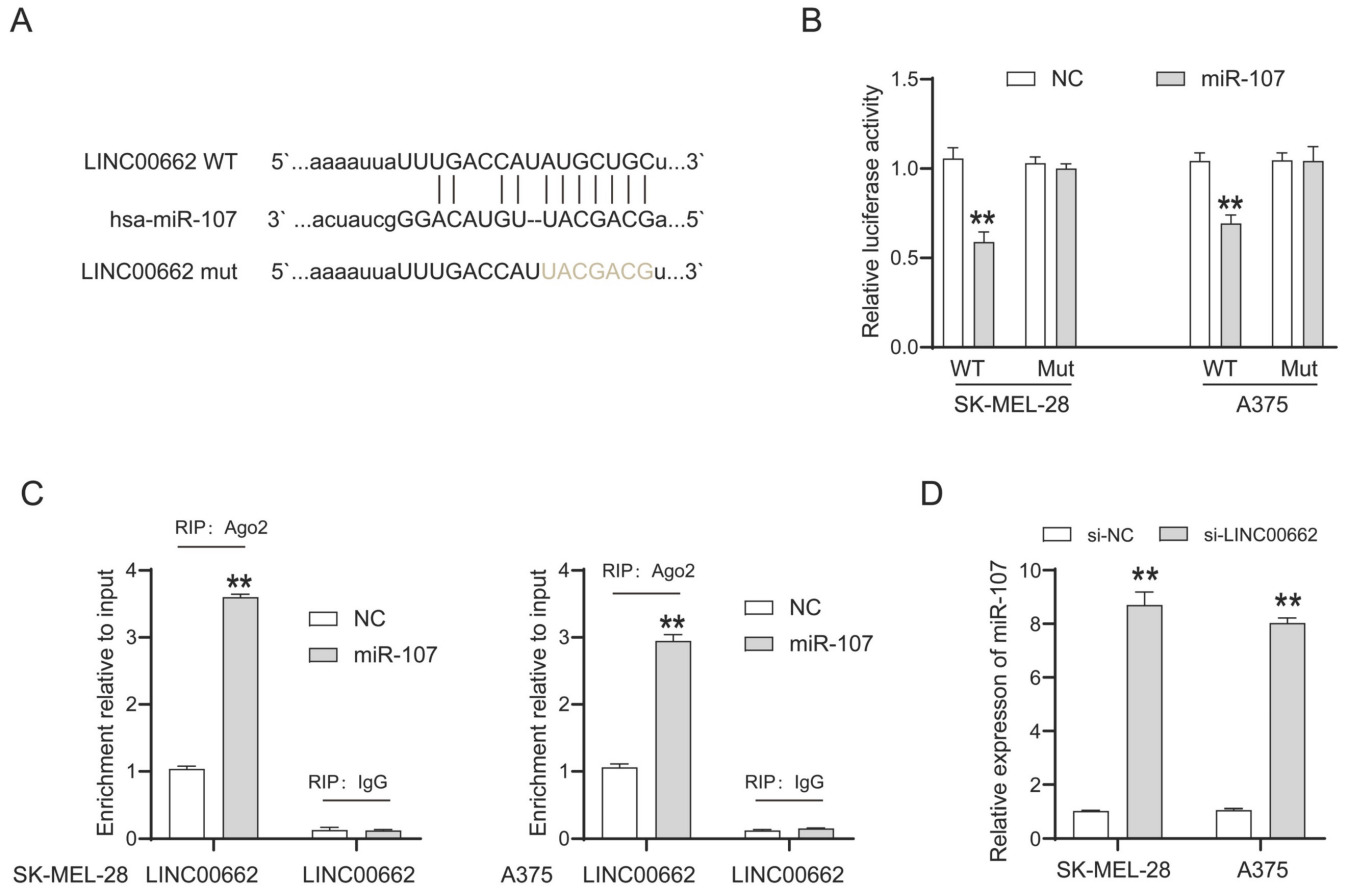
miR-107 was an essential downstream target of LINC00662 in melanomas. (A) The potential binding sites between miR-107 and LINC00662. (B) Luciferase reporter assays were performed to verify the binding activity between LINC00662 and miR-107. (C) RIP assay indicated that LINC00662 was substantially enriched by miR-107 overexpression with anti-Ago2 in SK-MEL-28 and A375 cells. (D) The expression level of miR-107 in SK-MEL-28 and A375 cells was significantly increased by knocking down LINC00662. **p<0.01.

LncRNAs cannot directly regulate cellular function but act as sponges of miRNAs. We searched for the potential target miRNAs of LINC00662 via online databases (RNA22 and miRcode). MiR-107 was predicted to be a promising target of LINC00662, and their binding sites are shown in Figure 4A. The luciferase reporter assay was used to verify the interaction between LINC00662 and miR-107. The luciferase activity in LINC00662-WT was significantly decreased by miR-107 mimic and no significant changes were observed in other groups (Figure 4B). The decreased fluorescence activity in the LINC00662-WT group indicated that the miR-107 was a direct target gene of LINC00662. Furthermore, a RIP assay was used to detect the potentially endogenous interaction between LINC00662 and miR-107. The data indicated that LINC00662 was substantially enriched by miR-107 overexpression with anti-Ago2 in SK-MEL-28 and A375 cells (Figure 4C). The expression level of miR-107 in melanoma cells was detected by RT-qPCR, and the results showed that silencing of LINC00662 significantly increased the expression level of miR-107 in SK-MEL-28 and A375 cells (Figure 4D). Additionally, the expression level of miR-107 was significantly increased by treatment with the miR-107 mimic and decreased by treatment with the miR-107 inhibitor in SK-MEL-28 and A375 cells (Fig. S2). Together, the results suggested that miR-107 was an essential target gene of LINC00662 in melanoma.

### LINC00662 affected melanomas through miR-107/POU3F2

**Figure 5:**
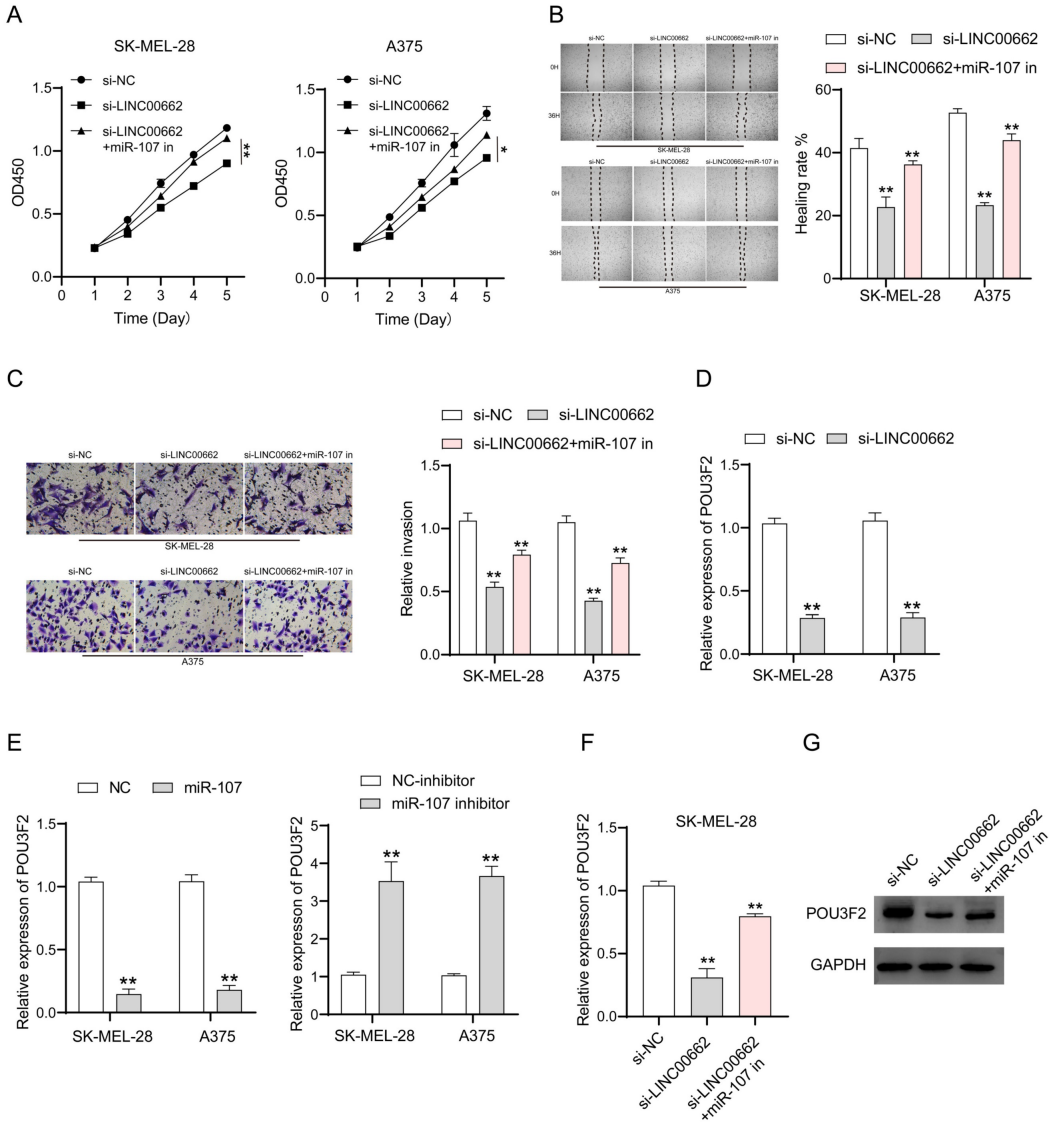
** LINC00662 affected melanomas through miR-107/POU3F2.** (A) CCK-8 assays demonstrated that miR-107 inhibitor partially counteracted the si-LINC00662-induced reduction in cell proliferation. (B-C) Wound healing and Transwell assays showed that mir-107 inhibitor abolished the effects of si-LINC00662 on cell migration and invasion. (D) The expression level of POU3F2 was reduced after silencing the expression of LINC00662 in SK-MEL-28 and A375 cells. (E) The expression levels of POU3F2 in SK-MEL-28 and A375 cells transfected with miR-107 mimic, inhibitor, or its corresponding negative control. (F-G) q-RTPCR and Western blot analysis were implemented to analyze the RNA and protein levels of POU3F2 in SK-MEL-28 cells after transfected with si-NC, si-LINC00662 or si-LINC00662+miR-107 inhibitor. *P<0.05, **P<0.01.

To verify whether the involvement of LINC00662 in melanoma is realized through miR-107, we co-transfected si-LINC00662 and the inhibited miR-107 mimics in SK-MEL-28 and A375 cells. CCK-8, wound healing assay, and Transwell assays were used again to detect changes in cell function. Cell proliferation, cell migration, and invasion are inhibited by silencing LINC00662, and it was thought that a miR-107 inhibitor may rescue this si-LINC00662-mediated inhibition of cell function. As expected, the effect on cell function by knocking down LINC00662 was partly abrogated by using a miR-107 inhibitor. CCK-8 assays demonstrated that miR-107 inhibitor partially counteracted the si-LINC00662-induced reduction in cell proliferation (Figure 5A). Wound healing and Transwell assays showed that miR-107 inhibitor abolished the effects of si-LINC00662 on cell migration and invasion (Figure 5B and C). Therefore, we proved that miR-107 was involved in melanoma progression as a tumor suppressor and was regulated by LINC00662. In a previous report, miR-107 acted as a tumor suppressor by targeting POU3F2 19, thus, we hypothesized that POU3F2 was involved in the LINC00662/miR-107-dependent pathway in melanoma. To further confirm this hypothesis, we measured the expression level of POU3F2 in melanoma cells by RT-qPCR. The results showed that silencing of LINC00662 significantly decreased the expression level of POU3F2 in SK-MEL-28 and A375 cells (Figure 5D). We also found that the expression level of POU3F2 was significantly decreased by treatment with miR-107 mimic and increased by treatment with the miR-107 inhibitor (Figure 5E). Next, we co-transfected si-LINC00662 and the miR-107 mimics inhibitor in SK-MEL-28 cells and detected the expression of POU3F2 by qPCR and western blotting. The results showed that POU3F2 was downregulated when LINC00662 was silenced, whereas the si-LINC00662-induced down-regulation of POU3F2 expression was reversed when it coexisted with the miR-107 mimic inhibitor (Figure 5F). The protein level of POU3F2 was markedly decreased by silencing LINC00662, which was reversed by the miR-107 mimics inhibitor (Figure 5G). Collectively, these results indicated that LINC00662 acted as a ceRNA to regulate POU3F2 expression by sponging miR-107 in melanomas.

### POU3F2 overexpression attenuated the effect of LINC00662 knockdown on the development of melanomas

**Figure 6:**
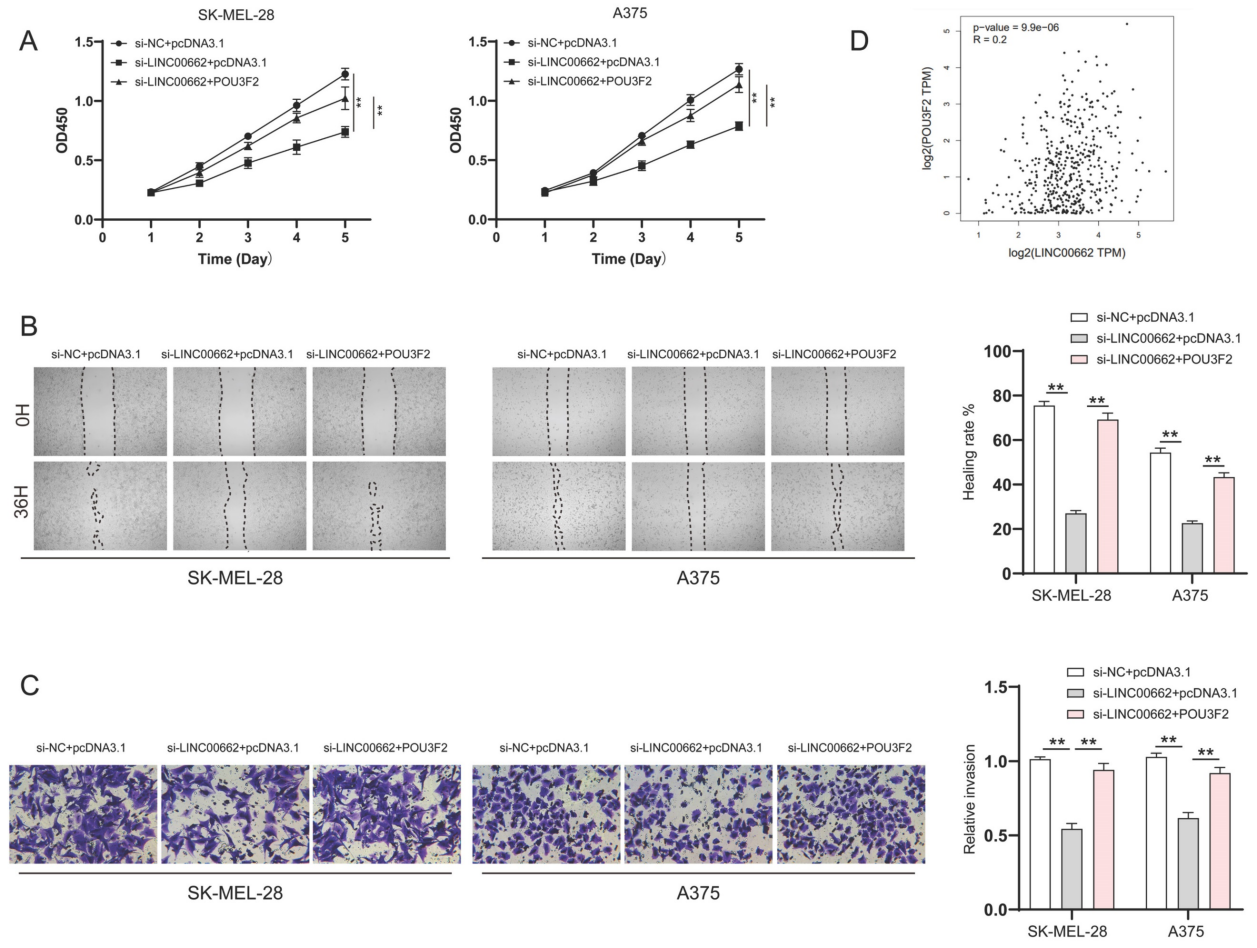
POU3F2 overexpression attenuated the effect of LINC00662 knockdown on the development of melanomas. (A) CCK-8 assays were implemented to detect cell proliferation upon co-transfection of si-LINC00662 and pcDNA3.1- POU3F2 plasmid. (B-C) Wound healing (magnification, x100) and Transwell assays (magnification, x200) showed that miR-107 inhibitor abolished the effects of si-LINC00662 on cell migration and invasion. (D) The TCGA data showed a positively correlation between LINC00662 and POU3F2 (R=0.2, P<0.01). **P<0.01.

To verify whether LINC00662 played a role in melanoma progression through POU3F2, SK-MEL-28 and A375 cells were co-transfected with si-LINC00662 and the pcDNA3.1- POU3F2 plasmid. The CCK-8 wound healing and Transwell assays were again used to detect changes in cell function. The results suggested that overexpressing POU3F2 reduced the effect of si-LINC00662 on cell functions, including cell viability, migration, and invasion (Figure 6A-C). In addition, correlation analysis revealed that POU3F2 positively correlates with the expression of LINC00662 in melanoma (Figure 6D). Overall, these results confirmed that POU3F2 was a downstream target of LINC00662/miR107, and that LINC00662 participated in the development of melanomas by binding miR-107 and activating POU3F2.

### The β-catenin signaling was involved in the LINC00662-induced function in melanomas

**Figure 7:**
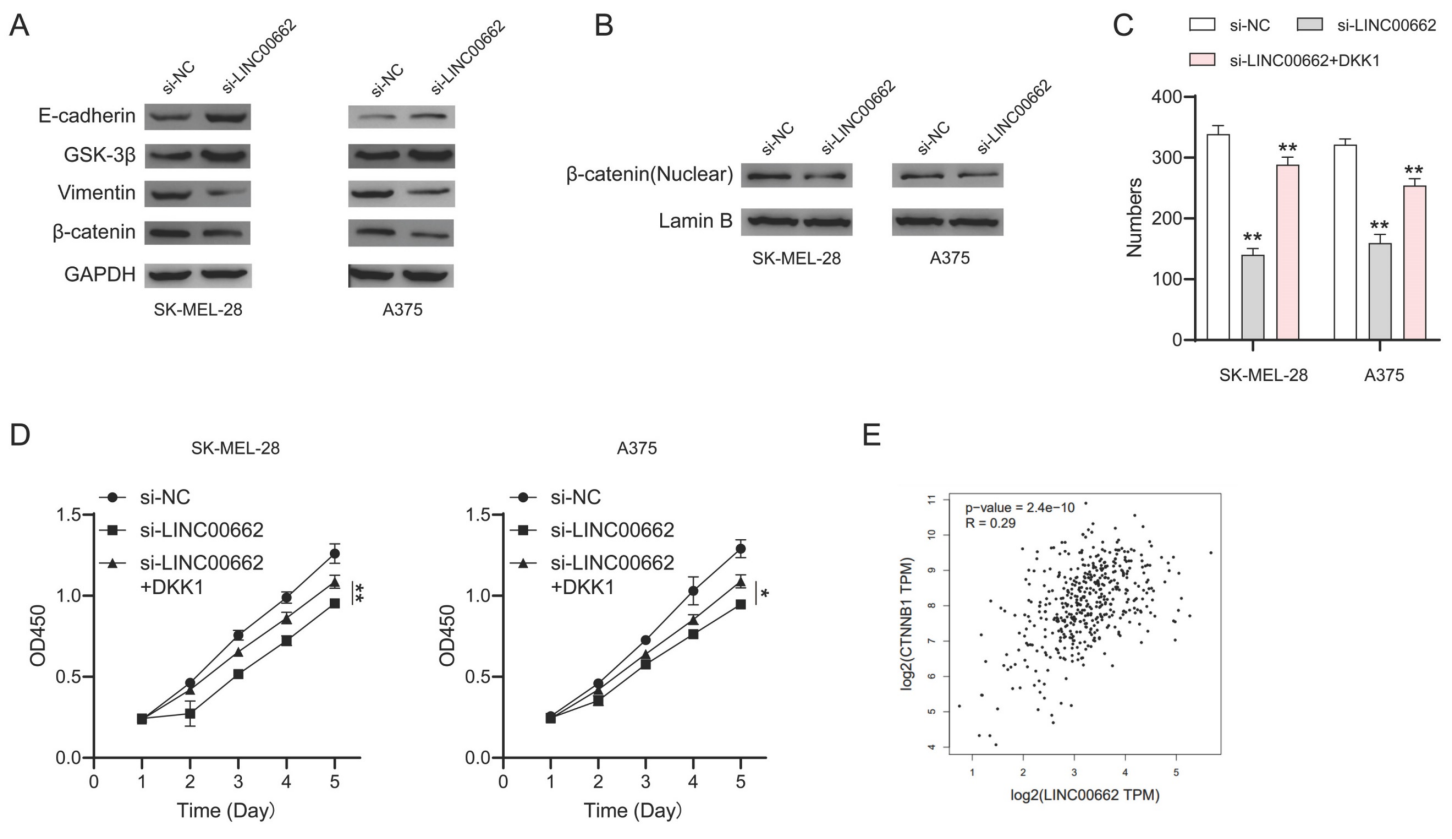
** β-catenin signaling was involved in LINC00662-induced function in melanomas.** (A) The expression levels of E-cadherin, GSK-3β, vimentin, and β-catenin were detected by Western Blot analysis. (B) si-LINC00662 reduced the levels of β-catenin protein in the nucleus. (C-D) CCK-8 and colony formation assays were used to detect cell proliferation after transfection with si-LINC00662 or si-LINC00662 + DKK 1, and si-NC was used as a control. (E) The TCGA data showed a positively correlation between LINC00662 and β-catenin (R=0.29, P<0.01). *P<0.05, **P<0.01.

To further investigate the mechanism of LINC00662, we focused on β-catenin signaling which plays an important role in melanoma. We performed a western blotting assay to test the expression of the key roles in β-catenin signaling. We found that E-cadherin and GSK-3β were up-regulated while vimentin and β-catenin were downregulated in si-LINC00662 melanoma cells (Figure 7A). Meanwhile, si-LINC00662 can also reduce the levels of β-catenin protein in the nucleus (Figure 7B). These findings suggested that the knockdown of LINC00662 inactivated β-catenin signaling. Subsequently, we performed rescued experiments in si-LINC00662 cells treated with or without the β-catenin agonist DKK-1, both colony formation and CCK-8 assay showed that DKK-1 partly abolished the effect of silencing LINC00662 (Figure 7C and D). Moreover, correlation analysis revealed that β-catenin positively correlates with the expression of LINC00662 in melanoma (Figure 7E). All the results revealed that β-catenin signaling was involved in a LINC00662-induced function in melanoma.

## Discussion

This study is the first to investigate the role of the LINC00662-miR-107-POU3F2 axis in melanoma development and its underlying mechanisms. We reported that the expression of LINC00662 was high in melanomas. Silencing LINC00662 not only inhibited cell proliferation, cell migration, and invasion *in vitro,* but also inhibited tumor growth *in vivo*, while LINC00662 overexpression promoted melanoma cell proliferation, suggesting that LINC00662 had an oncogenic effect in melanomas. Furthermore, our study demonstrated that miR-107 was involved in melanoma progression as a tumor suppressor and was regulated by LINC00662. Our study also showed that silencing LINC00662 decreased the expression of POU3F2 by competitively inhibiting its binding to miR-107, confirming that LINC00662 served as a ceRNA to target POU3F2 by sponging miR-107 and LINC00662, which may promote melanoma development by upregulating miR-107-mediated POU3F2 expression.

LINC00662 is a functional lncRNA that has been reported in multiple tumors. The various functional mechanisms and carcinogenesis of LINC00662 suggest that it is broadly involved in many aspects of cancer 21-26. However, few articles have reported the role of LINC00662 in melanoma, and so far, we have found only one previous report of LINC00662 in melanomas, which demonstrated that LINC00662 promoted cell proliferation, migration, and invasion in melanomas by upregulating ELK3 by sponging miR-890 18. Consistently, our study showed that LINC00662 was highly expressed in melanoma patients, and high expression of LINC00662 in melanomas was associated with a poor patient prognosis. Our findings suggested that LINC00662 may serve as a potential diagnostic biomarker for melanomas. The silencing of LINC00662 *in vitro* inhibited the proliferation, migration, and invasion of melanoma cells. In contrast, overexpressing LINC00662 promoted melanoma cell proliferation *in vitro*. In addition, we performed *in vivo* tumorigenesis experiments in nude mice. We found that the suppression of LINC00662 reduced xenograft growth in animal experiments. All of these findings suggested that LINC00662 was an oncogene in melanomas, and that it may act as a marker.

MiRNAs are involved in the regulation of cellular processes as post-transcriptional regulators that target the 3´-UTR of mRNAs 27. One miRNA can regulate multiple potential target genes, and recent studies have shown that miRNAs are also regulated by lncRNA. LncRNA itself is not capable of encoding proteins but works by binding miRNAs in a process known as the competitive endogenous theory. Similarly, ceRNA has been widely recognized in cancer research, for example, lncRNA MNX1-AS1 promotes laryngeal squamous cell carcinoma progression and serves as a ceRNA to target FoxM1 by sponging miRNA-370 28. Hypoxia-induced lncHILAR promotes renal cancer metastasis via ceRNA in the miR-613/206/1-1-3p/jagged-1/Notch/CXCR4 signaling pathway 29. In melanoma, lncRNA MSC-AS1 promotes the pathological process by binding to miR-302a-3p 30, lncRNA MIAT can promote proliferation, migration, and invasion in melanoma through recruiting TCF12 31, and lncRNA HCG18 facilitates melanoma progression by modulating the miR-324-5p/CDK16 axis 32.

In recent years, LINC0662 has been reported to bind to numerous miRNAs and regulate tumor progression. In the present study, we hypothesized that miRNAs capable of binding LINC0662 included miR-107^17^, miR-340-5p 16, and miR-144-3p 22. Increasing evidence has reported that miR-107 functions as a tumor suppressor in numerous cancers, including renal clear cell carcinomas, hepatocellular carcinomas, colorectal cancers, and breast cancers 33-36. Notably, a recent study reported that miR-107 was significantly downregulated in metastatic melanomas, so miR-107 may have a tumor suppressor function in metastatic melanomas 19. We chose to focus on miR-107 because of the specificity of miR-107 expression in melanomas, as well as the lack of related studies, and because LINC00662 was positively correlated with the expression of POU3F2, a downstream target of miR-107.

Our study provided clear evidence that LINC00662 functioned as a sponge for miR-107, which was confirmed as a target miRNA of LINC00662. Bioinformatics analysis showed that there were binding sites between LINC00662 and miR-107, which were verified by the luciferase reporter and RIP assays. RT-qPCR results showed that the expression of miR-107 was significantly increased when LINC00662 was silenced. The miR-107 inhibitor partially abrogated the effect of silencing LINC00662 in melanoma cell function. These results suggested that miR-107 played an integral role in the tumor-promoting effect of LINC00662 in melanomas. One lncRNA can bind to multiple miRNAs. For example, LINC00662 has been reported to interact with miR-890 in melanomas 18, which does not contradict our results and suggests that the functional mechanisms of LINC00662 are diverse in melanomas and require further investigation. In addition to competitively targeting the expression of miR-107 and then regulating the downstream gene POU3F2, whether LINC00662 has another mechanism of action in melanoma to regulate tumor progression requires further verification. Therefore, we hypothesized that there is a possibility that LINC00662 could act through additional miRNAs and further experiments are underway.

Notably, our study identified *POU3F2* as a downstream gene of the LINC00662/miR-107 axis in melanoma. RT-qPCR and western blot assays showed that POU3F2 was downregulated when LINC00662 was silenced; moreover, the effect of silencing LINC00662 on POU3F2 expression was partially abolished by the miR-107 mimic inhibitor. The negative effect of silencing LINC00662 on melanoma cell proliferation and migration was partially abrogated by miR-107 mimetic inhibitors or POU3F2 overexpression. Thus, our study suggested that the LINC00662/miR-107/POU3F2 axis may play an important role in melanoma progression. In the future, we intend to validate its effect in melanomas* in vivo* using xenograft or UV-induced mouse models.

Accumulated evidence suggests that the β-catenin signaling pathway plays an important role in the development of cancer. In this study, we demonstrated that the β-catenin signaling pathway was involved in LINC00662-induced function in melanoma. E-cadherin and GSK-3β were up-regulated whereas vimentin and β-catenin were downregulated in si-LINC00662 melanoma cells, and si-LINC00662 can also reduce the levels of β-catenin protein in the nucleus which might affect β-catenin transcriptional activity. Moreover, CCK-8 and colony formation assays indicated that the β-catenin agonist, DKK-1, reversed the si-LINC00662-induced reduction in cell proliferation. LINC00662 may also affect other pathways, but the current results suggest that the β-catenin signaling pathway is involved in LINC00662-induced function. There are some reports about the relationship between miR-107 and β-catenin 37-39 which further confirm our conclusion. It is possible that LINC00662 can act through other pathways as well, and further research will be carried out in the future.

## Conclusion

Our study was focused on the function of LINC00662 in melanomas and has demonstrated that miR-107 was the downstream target of LINC00662. Our findings suggested that the LINC00662/miR-107/ POU3F2 axis may be one of the ways that LINC00662 promotes melanoma growth and metastasis. Our study revealed a novel ceRNA regulatory pathway through the competitive endogenous binding of miR-107 and activation of POU3F2. Furthermore, the β-catenin signaling pathway was confirmed to be involved in the LINC00662-induced effect.

## Supplementary Material

Supplementary figures and table.
