# Supplementary material for: LINC00662 promotes melanoma progression by competitively binding miR-107 and activating the β-catenin signaling pathway
Source: Int J Med Sci. 2024 Jan 1;21(2):265–76. doi: 10.7150/ijms.84072 (PMC10758139; doi:10.7150/ijms.84072)
Supplement: Supplementary file 1 — Supplementary figures and table. [file ijmsv21p0265s1.pdf]

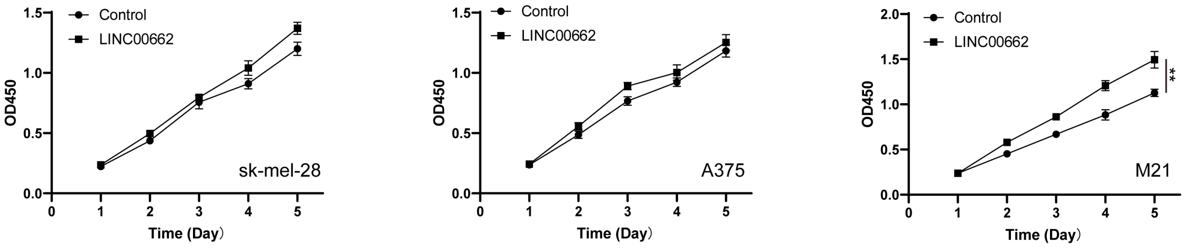

Fig. S1. CCK-8 assays demonstrated that overexpression of LINC00662 slightly promoted cell proliferation in SK-MEL-28 and A375 cells. Cell proliferation was significantly increased due to LINC00662 overexpression in M21 cells.

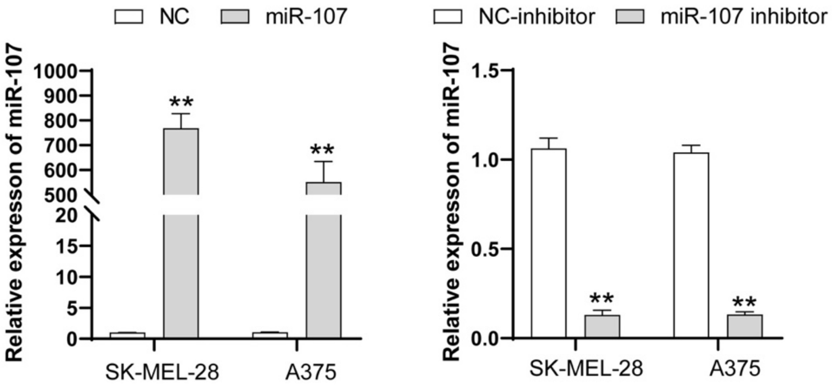

Fig. S2. The expression level of miR-107 was significantly increased by treatment with the miR-107 mimic and decreased by treatment with the miR-107 inhibitor in SK-MEL-28 and A375 cells.

Table S1. Primer sequences used for RT-qPCR and cell transfection.

Targets	Name	Sequence
Primers used for RT-qPCR.		
LINC00662	F:	5'-TGGACATCTGTCTGGAGG-3'
	R:	5'-GGCTGAGGCATAAGAATCG-3'
miR-107	F:	5'-ATGATGAGCAGCATTGTACAGG-3'
	R:	5'-GCAGGGTCCGAGGTATTC-3'
POU3F2	F:	5'-GAAGTGTCCAATCAGTGTTGG-3'
	R:	5'-GTCAGTGTTCCAAGAAGGAC-3'
β-actin	F:	5'-GGGAAATCGTGCGTGACATTAAG-3'

	R:	5'-TGTGTTGGCGTACAGGTCTTTG-3'
GAPDH	F:	5'-GGAGCGAGATCCCTCCAAAAT-3'
	R:	5'- GG CTGTTGTCATACTTCTCATGG -3'
U6	F:	5'-GCTTCGGCAGCACATATACTAAAAT-3'
	R:	5'-CGCTTCACGAATTTGCGTGTCAT-3'
Primers used for cell transfection.		
si-LINC00662-1		AAUGUACACUUAUUCAUUCCA
si-LINC00662-2		AUACAAAGACCGUUCAAUGGG
si-NC		UUCUCCGAACGUGUCACGUUU
miR-107 mimics		AGCAGCAUUGUACAGGGCUAUCA
miR-107 inhibitor		UGAUAGCCCUGUACAAUGCUG
miR-NC mimics		UUGUACUACACAAAAGUACUG

Note: F: Forward primer; R: Reverse primer. GAPDH, glyceraldehyde-3-phosphate dehydrogenase;
